# Patient Characteristics in Ulnar Nerve Compression at the Elbow at a Tertiary Referral Hospital and Predictive Factors for Outcomes of Simple Decompression versus Subcutaneous Transposition of the Ulnar Nerve

**DOI:** 10.1155/2019/5302462

**Published:** 2019-12-19

**Authors:** Alice Giöstad, Erika Nyman

**Affiliations:** ^1^Division of Clinical Sciences, Department of Clinical and Experimental Medicine, Linköping University, Linköping, Sweden; ^2^Department of Hand Surgery, Plastic Surgery and Burns and Department of Clinical and Experimental Medicine, Linköping University, Linköping, Sweden

## Abstract

Patient characteristics and predictive factors for outcomes were analysed in 202 cases undergoing simple decompression, primary subcutaneous transposition, or secondary subcutaneous transposition for ulnar nerve compression at the elbow at a tertiary referral hospital. Data from medical charts and a survey were evaluated. The mean patient age was 49 years with revision surgery cases being significantly younger. Sixty-one percent of cases were female, and 31% were smokers. The comorbidity was extensive, including other nerve compression lesions as well as neck and shoulder problems. Overall, 53% reported being pleased with the result of surgery and 57% of the cases rated function as better or completely recovered after surgery. The median postoperative DASH (Disabilities of the Arm, Shoulder and Hand) score was 26 (IQR 11–49), which is in accordance with unpublished national data. No significant differences in DASH scores were found between surgical groups, but a higher preoperative McGowan grade was significantly associated with a poorer postoperative DASH score. Women scored greater disability postoperatively than men. There was a significantly increased risk of complications, which was doubled for smokers, following primary and secondary transposition compared to simple decompression. Surgical cases with ulnar nerve compression treated at a tertiary referral hospital constitute a heterogeneous group with great comorbidity and frequent concomitant nerve compression lesions. We suggest simple decompression as the procedure of first choice. Transposition can be used in selected cases or when simple decompression fails. All patients should be strongly recommended to stop smoking considering the remarkably increased risk for complications among smokers.

## 1. Introduction

Ulnar nerve compression is a relatively common peripheral nerve compression disorder in the upper extremities, but the outcome is not as predictable as for carpal tunnel syndrome [1, 2]. Numbness and paraesthesia in the affected arm and hand are early symptoms. If the condition deteriorates, atrophy of the innervated muscles with resulting loss of motor function, claw hand deformity, and even severe pain may occur. The diagnosis of ulnar nerve compression is typically based on the patient's history and a clinical examination. Electrophysiology is frequently used, but its value for diagnosis and outcomes is debated [3]. If conservative noninvasive treatment fails, surgery is indicated [4]. Many patients are handled by orthopedic surgeons at provincial hospitals, while more complex cases are referred to specialized units with competence in nerve surgery. Several surgical options are available, but there is currently no consensus on which method to use when giving treatment for ulnar neuropathy at the elbow [5]. In particular, the literature is inconclusive regarding factors predicting surgical outcomes, especially concerning cases at referral hospitals, where additional, complicated factors may be relevant.

At the Department of Hand Surgery, Plastic Surgery and Burns, Linköping University Hospital, Linköping, Sweden, simple decompression and anterior subcutaneous transposition are used as surgical treatments for ulnar nerve compression at the elbow. Revision surgery, with ulnar nerve transposition after simple decompression, is a well-known solution for persistent or recurrent symptoms [6]. Several meta-analyses comparing these surgical methods have failed to reveal statistically significant differences in outcomes [7–10]. Chen et al., and Said et al., suggested that simple decompression should be preferred due to the lower frequency of complications compared to anterior subcutaneous transposition of the ulnar nerve, while Macadam et al. found some evidence for an improved clinical outcome with anterior transposition [7, 8, 11] In addition, Lauretti et al., in a systematic review, found that two out of 24 studies had a lower complication rate for simple decompression than anterior transposition and that no significant difference in satisfactory outcomes was seen between the methods [11].

The main objectives of this study were to study patients' characteristics in ulnar nerve compression at a tertiary referral hospital and to analyse predictive factors for outcomes of simple decompression versus subcutaneous transposition of the ulnar nerve.

## 2. Methods

Ethical approval was obtained from the Regional Ethics Review Board in Linköping, Sweden (register number 2016/88-31). A retrospective observational study was performed on all patients, with no exclusions, having surgery for ulnar nerve compression at the elbow at the Department of Hand Surgery, Plastic Surgery and Burns, Linköping University Hospital, Linköping, Sweden, which is a tertiary referral hospital, between 1 January 2011 and 31 December 2014. No cases related to trauma, such as fractures or luxation of the elbow, were treated at this unit. The data were obtained from patient charts and from questionnaires sent out to the patients. The minimum follow-up time was 12 months.

The medical records were coded using two numbers: a patient number and a case number for the surgery. If the patient had bilateral surgeries, each arm was given a case number. Background data collected from the patient charts were age, gender, smoking habits, blue-collar work, comorbidity, use of pharmaceutical drugs, affected dominant arm, other nerve compression lesions in the same/opposite arm, preoperative McGowan grade [12], and whether conservative treatment was given. The surgical method, time between primary surgery and possible revision surgery, complications (e.g., emergent neurogenic pain after surgery, affection/loss of nerve function, including hypoesthesia around the area of surgery, postoperative infection, postoperative bleeding, or complex regional pain syndrome (CRPS) in conjunction with the surgery), results from electrodiagnostic testing (affection of the ulnar nerve or not), and magnetic resonance imaging (MRI) (nerve root affection or not) were also noted.

The Disabilities of the Arm, Shoulder and Hand (DASH) outcome questionnaire [13] and a specially designed form ([Supplementary-material supplementary-material-1], modified from Svernlöv et al. [14]) were sent out by mail, together with information about the study and a written consent form, to all patients, apart from those who were deceased or unable to understand written Swedish, at the time of the study. Patients who did not respond to the letter were sent a postal reminder, and any remaining nonresponders were contacted by telephone. The specially designed form included questions regarding symptoms pre- and postoperatively, function, and patient satisfaction.

There were 173 patients enrolled, including 29 with bilateral surgery, resulting in a total of 202 cases being reviewed. Cases were divided into three groups according to the surgical interventions used at the unit, i.e., simple decompression, primary anterior subcutaneous transposition, and secondary anterior subcutaneous transposition (revision surgery after simple decompression due to persistent or recurrent symptoms). Each case could only be included in one group, and there was no cross-over between groups.

### 2.1. Statistical Analyses

Data are presented as mean ± SD or median (IQR). Nonparametric tests were generally used in order to evaluate any differences among the three surgical interventions. The Shapiro–Wilk test of normality was used to control for normal distribution of data. One-way ANOVA with the Bonferroni correction was used to test for differences in mean age at the time of the first surgery. Differences in categorical background data were analysed using chi-square statistics. If this was not applicable, Kruskal–Wallis tests with subsequent Mann–Whitney *U* test to identify differences between separate groups when significant were used. Differences in continuous, normally distributed background data were calculated by means of various parametric tests. Logistic regressions were used to assess the risk of complications and evaluate associated factors for patient-reported satisfaction after surgery. Binary logistic regression was used when testing whether the type of surgery influenced patient satisfaction and self-reported function of the arm/hand. Postoperative DASH scores were compared between patients with normal or pathological results from electrophysiology using the Mann–Whitney *U* test. The threshold for significance in all tests was set at a *p* value <0.05. Collected data were aggregated and analysed at the group level. The choice of statistical methods and analysis of the results were discussed with a professional statistician.

## 3. Results

### 3.1. Medical Charts

Fifty-six percent (*n* = 114) of the cases had simple decompression, 28% (*n* = 56) had primary anterior subcutaneous transposition, and 16% (*n* = 32) had secondary anterior subcutaneous transposition. The choice of surgical intervention was based on patient history, clinical examination, such as Tinel's sign or palpation for tenderness and subluxation of the ulnar nerve, and the result of electrophysiology according to the medical charts. The main documented reason for the surgeon's choice of primary transposition was pre- or peroperative subluxation of the nerve or a history of revision surgery with secondary transposition in the contralateral arm due to a poor initial outcome after simple decompression. The preoperative McGowan grade was equally distributed between surgery groups and was not associated with patient-reported satisfaction after surgery (chi-square statistics, *p*=0.32 and *p*=0.62, respectively; Tables 1 and 2). The time lapse between the simple decompression and the secondary transposition of the ulnar nerve varied from 6 to 324 months (median 16 months, IQR 10–33).

The mean age for the whole population was 49 ± 14 years. Cases with secondary ulnar nerve transposition were significantly younger than those who were treated with simple decompression (one-way ANOVA, *p*=0.013; Bonferroni correction, *p*=0.014). Sixty-one percent (123/202) of the cases in the study population were female, but there was no significant difference in gender distribution among the three surgical groups. Signs of compression of the median or the radial nerves in the same arm were seen in 54% (109/202) of the cases and in 35% (70/202) in the contralateral arm. No statistically significant difference between another nerve compression lesion and the type of surgical intervention was shown by chi-square statistics. The mean body mass index (BMI) was 27, 25% (50/202) had neck problems, 16% (33/202) had shoulder problems, and 13% (26/202) had diabetes. No significant differences were found between these variables and the type of surgery (Table 3 shows selected background data).

The logistic regression showed that a higher age was associated with an increased likelihood of being displeased with the results after surgery (Exp(B) = 1.03, 95% CI 1.00–1.07, *p*=0.040), while no other associations were found for the investigated variables (age, gender, and preoperative McGowan grade).

Thirty-one percent (63/202) of the cases were smokers at the time of surgery. There was no statistically significant difference in smoking habits between the patients included in the three surgical interventions. The logistic regression, however, showed a statistically significant difference regarding complications and smoking habits with the risk of complications being more than twice as high for smokers (Exp(B) = 2.23, 95% CI 1.09–4.57, *p*=0.029).

**Figure 1:**
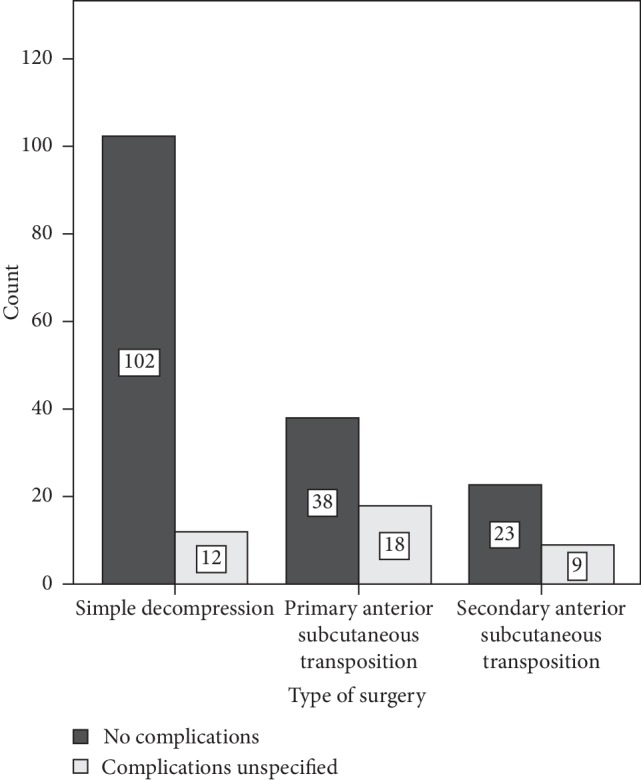
Distribution of complications in 202 cases with surgery for ulnar nerve compression at the elbow. Eighty-one percent (163/202) of the surgeries were completed without any complications. The complication rate was 11% (12/114) for simple decompression, 32% (18/56) for primary anterior subcutaneous transposition, and 28% (9/23) for secondary anterior subcutaneous transposition.

Complications were seen in 19% (39/202) of the cases. The occurrence and distribution of complications can be seen in Figure 1 and Table 4. Emergent neurogenic pain after surgery was the most common complication (8%, 16/202), followed by affection/loss of nerve function around the surgery area, such as hypoesthesia (7%, 15/202). The complication rate was significantly increased (Exp(B) = 1.97, 95% CI 1.26–3.08, *p*=0.003) when transposition was used compared to simple decompression, and complications were most common after primary transposition.

### 3.2. Surveys

In total, 168 surveys were sent out and the response rate was 61%, with a similar response rate for each surgical intervention. There were no differences in age, gender, or type of surgery between the responders and the nonresponders. The observed distribution differed significantly from what was expected regarding the preoperative McGowan grade (chi-square statistics, *p* < 0.001), where more patients with McGowan grade 3 and fewer patients with grade 1 answered the questionnaires.

There was a variation in the total number of cases for the different questions and the DASH questionnaire as all questions were not correctly answered by the patients. Regarding experience of reduction in grip strength pre- and postoperatively and VAS-estimated pain during activity preoperatively, there were significant differences between the three intervention groups before surgery (Table 1 shows patient-reported pre- and postoperative symptoms). There were no significant differences among the various surgical interventions with respect to grading of the other self-reported pre- and postoperative symptoms, such as estimated pain at rest (VAS), occurrence of paraesthesia, perceived reduction in sensitivity, occurrence of claw hand deformity, ability to abduct/adduct fingers, or sleeping difficulties.

The symptom that showed the greatest improvement was paraesthesia, where 56% of the population (64% for simple decompression, 44% for primary transposition, and 50% for secondary transposition) experienced a relief of symptoms and 6% a worsening of symptoms. Motor symptoms did not improve as much as the sensory ones. The symptom with the worst outcome was reduction in grip strength. Concerning sleeping difficulties, 58% of the patients had fewer sleeping disturbances after surgery than before surgery (pre- and postoperative symptoms are presented in Table 1).

**Figure 2:**
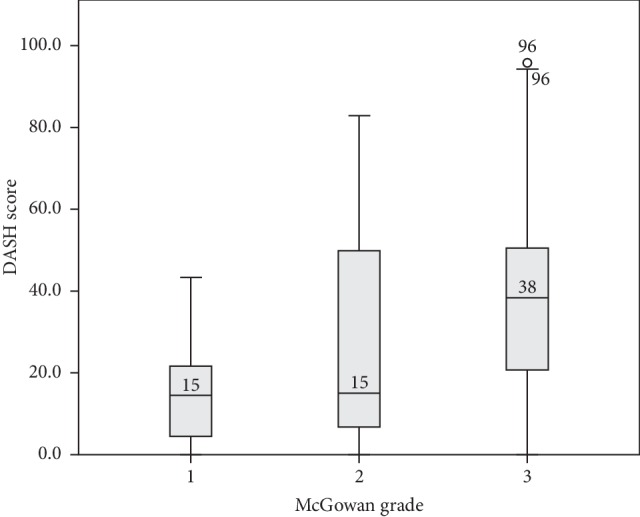
Postoperative scores from the Disabilities of the Arm, Shoulder and Hand (DASH) questionnaire and the distribution for each preoperative McGowan grade. The median value for each McGowan grade is a marked line on the box plot. Grade 3 statistically differed from grades 1 and 2 (Kruskal–Wallis test, *p*=0.002; subsequent Mann–Whitney *U* test; values written in McGowan grade 3 are outliers).

**Figure 3:**
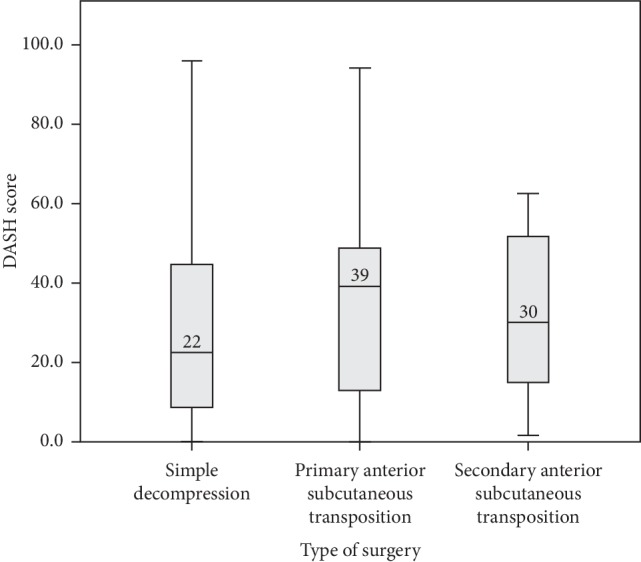
Disabilities of the Arm, Shoulder and Hand (DASH) scores calculated for 103 cases treated with different surgical interventions (simple decompression, *n* = 58; primary transposition group, *n* = 31; secondary transposition, *n* = 14) for ulnar nerve compression at the elbow. The results varied from zero to 96. The median value for each surgical intervention is a marked line on the box plot (Kruskal–Wallis test, *p*=0.36).

In total, DASH scores from 103 cases could be calculated from the surveys (58 from the simple decompression group, 31 from the primary transposition group, and the remaining 14 from the secondary transposition group). The median postoperative DASH score for the whole study population was 26 (IQR 11–49), with scores being 22 (IQR 8–47) for simple decompression, 39 (IQR 9–49) for primary transposition, and 30 (IQR 15–52) for secondary transposition. The Shapiro–Wilk test of normality was significant, showing that the variable DASH score was not normally distributed. There was a statistically significant difference in the postoperative DASH score with respect to preoperative evaluation with the McGowan grade (Kruskal–Wallis test, *p*=0.002) with a significant difference in the DASH score between McGowan grades 1 and 3 as well as 2 and 3 (Mann–Whitney *U* test; Figure 2). Women reported significantly higher postoperative DASH scores than men (Mann–Whitney *U* test, *p*=0.026). There were no statistically significant differences among the surgical groups concerning the DASH score (Kruskal–Wallis test, *p*=0.36; Figure 3).

A majority (53%, 55/103) of the cases reported being generally pleased or very pleased with the results of the surgery (53% (31/58) for simple decompression, 48% (15/31) for primary transposition, and 64% (9/14) for secondary transposition). Binary logistic regression showed no significant difference between the type of surgery and reported satisfaction. Regarding function, 57% (69/105) of the cases rated the hand/arm function as better or completely recovered at the time of the survey than before surgery (58% (34/59) for simple decompression, 53% (17/32) for primary transposition, and 64% (9/14) for secondary transposition). Binary logistic regression showed no significant difference between the type of surgery and reported function. All those who answered “completely recovered” had had simple decompression. After a secondary transposition, 64% reported functional improvement and no case indicated an outcome that was worse. The majority (71%, 74/104) of all cases would have the same surgery again (75% (44/59) would have simple decompression, 59% (19/32) primary ulnar nerve transposition, and 57% (8/14) secondary ulnar nerve transposition). Eight percent (8/105) of all the cases answered that they would not go through the same procedure again, and 21% (22/104) were not sure whether they would or not (based on what they know today about the surgery and the period afterwards).

### 3.3. Electrophysiology and Magnetic Resonance Imaging (MRI)

Electrophysiology was performed before surgery in 89% (180/202) of cases. According to the charts, the result was not graded but interpreted as pathological or nonpathological. There was no statistically significant difference in median DASH scores regarding the results from electrophysiology (Mann–Whitney *U* Test, *p*=0.39), nor was there any significant difference in occurrence of affection between the surgery groups regarding the results from electrophysiology (chi-square statistics, *p*=0.09) (Table 3).

Diseases, such as nerve root affection, were evaluated using MRI when suspected by the surgeon because of neck pain or affection of several nerves in the extremity. Patients with a pathological MRI were referred to the department of neurosurgery. Magnetic resonance imaging was done in 48% (96/202) preoperatively, and signs of any affection of nerve roots were seen in 14% (29/202) according to the medical charts. Significant differences neither in performing the MRI (chi-square statistics, *p*=0.12) nor in the occurrence of affection of nerve roots (chi-square statistics, *p*=0.46) were seen among surgical groups (Table 3).

## 4. Discussion

In the present study, surgically treated cases with ulnar nerve compression at the elbow at a tertiary referral hospital were found to constitute a heterogeneous group with great comorbidity and often several other nerve compression lesions in the same or opposite arm as well as a wide variation in the surgical outcome. The outcome and complication rate were evaluated for two commonly used techniques for primary surgery (simple decompression and anterior subcutaneous transposition) and for revision surgery with transposition. According to the literature, primary subcutaneous transposition and primary submuscular transposition are, in most respects, considered equally effective surgical procedures regarding outcomes [15–17].

Other relevant studies have populations with an age distribution similar to that in our study, with a mean age of around 50 years [14, 18–21]. Adelaar et al. found no relationship between age and the postoperative result, while Leone et al. stated that the result of primary anterior intramuscular transposition is less good in patients younger than 50 years [15, 22]. According to Gaspar et al., being younger than 50 years of age is the only significant predictor for revision surgery after simple decompression [21]. Camp et al., in a material of almost 26,000 patients, found young age to be among the most significant risk factors for impaired outcome [23]. The present results show that patients with ulnar nerve compression in need of revision surger*y* are significantly younger than those who had simple decompression, showing that younger age is a predictor for revision surgery. Higher age was shown to be associated with an increased likelihood of being displeased with the final results of surgery.

The possibility cannot be ruled out that patients with double crush syndrome or multifocal neuropathy, and thus with an increased susceptibility to a further nerve compression lesion at another level [24], have been included in the material and might somehow explain why some patients were not helped by surgery. A substantial part of the population was displeased with the results of the surgery, and patients with a pathological MRI, i.e., any affection of nerve roots, were more displeased (data not shown). However, only 8% (8/105) of the whole population stated that they would not go through the procedure again. Svernlöv et al. showed a satisfaction rate and a willingness to repeat the same procedure that were almost identical to those in our findings [14]. This suggests that the surgery and the postoperative care somehow seem to benefit the patients. The surgical groups were assessed as having similar background data and preoperative severity of symptoms, apart from estimated pain during activity and experienced reduction in grip strength. The preoperative McGowan grade, i.e., grade 3 being different from grades 1 and 2, was associated with a higher postoperative DASH score, but not with postoperative patient satisfaction.

Women reported significantly higher postoperative DASH scores than men, but no statistical difference in the postoperative DASH score could be found among the three types of surgical intervention. This must be seen in relation to the fact that some patients with persistent symptoms after simple decompression were reoperated on with transposition (and thus are found in the secondary transposition group). The DASH scores are in agreement with unpublished data on QuickDASH scores (the shortened version of DASH considered comparable to DASH) from the Swedish National Registry for Hand Surgery (HAKIR; http://www.hakir.se, Zimmerman et al. to be published), revealing a postoperative QuickDASH score of 34 (IQR 14–55) (*n* = 267) at 12 months for patients with simple decompression and 45 (IQR 33–64) (*n* = 34) for patients with primary transposition.

In this study, 16% of the cases had revision surgeries with transposition after a simple decompression, which is a higher number than that is seen in some other studies. Goldfarb et al. found that only 7% had persistent symptoms after simple decompression and were successfully treated with secondary submuscular transposition [25], and according to Camp et al., just 1.4% needed revision surgery [23]. All current patients were followed up by the surgeon and a physiotherapist at the same centre, which might lead to the identification of more patients with persistent or recurrent symptoms after primary surgery.

Our complication rate is higher than that shown by Lauretti et al., where no surgical complications were noted following 60 anterior subcutaneous transpositions [11]. In the present study, complications occurred significantly more frequently for transpositions than for simple decompressions and were most common for primary transpositions. This was also seen in a prospective randomized controlled study carried out by Bartels et al. [18]. The most common complication in their study was sensory loss around the scar. They did not, however, consider any other aspect of pain than “elbow pain.” Neurogenic pain constitutes a considerable proportion of the complications reported in our study. The second most common complication in the present study was affection of nerve function, which included a variety of conditions, e.g., hypoesthesia around the area of surgery. We excluded no patients due to comorbidity or revision surgery, which is a common approach in many studies. The current patient cohort includes cases with several other nerve compression lesions, as well as pain conditions, referred from other units. CRPS that is diagnosed several weeks after surgery was also included as a postoperative complication.

Interestingly, the complication rate for secondary transposition was not significantly increased compared to that for primary transposition, as might have been expected due to fibrosis after the first surgery. Increased postoperative neurogenic pain was seen in fewer than 2% for simple decompression compared to almost 20% for primary transposition and 13% for secondary transposition. The underlying mechanism(s) might be that the greater trauma associated with relocating the nerve during transposition engenders neurogenic pain, arising from disturbances of the circulation around the ulnar nerve or a minimal trauma to the nerve with subsequent structural intraneural changes. One-third of the present patients were smokers, and they had a significantly increased risk of complications, possibly due to microcirculation disturbances. However, smoking was not a predictive factor for secondary transposition in contrast to the findings reported by Camp et al. [23].

Electrophysiology was used to confirm that the ulnar nerve was affected rather than to measure the outcome of surgery. The results of preoperative electrophysiology predicted neither the outcome nor the choice of surgery. Results from electrophysiology were only coded as an affected or unaffected ulnar nerve, since this is how the physicians utilized the test, according to the charts. No grading was used, which is a limitation as the results are not binary in reality. The literature regarding the use of electrophysiology, for example, for prognosis and management of disorders of peripheral nerves, is unconvincing [3] and emphasizes the need for more studies, including some with a prospective design, with grading of electrophysiology, for prediction of the outcome of surgery.

One weakness of this study is the retrospective study design. The strengths are a rather large sample size and an appropriate response rate to the surveys. All patients were operated on at one single referral hospital, according to the same two surgical methods, and followed the same rehabilitation protocol. One independent person (AG), who is not a surgeon at the clinic, evaluated all the patient charts. We used patient-reported outcome measures (PROM), which are considered even more reliable for evaluating and predicting outcomes, but data could have been further strengthened with complementary objective methods, such as grip strength, pinch strength, and two-point discrimination [26, 27]. However, such “objective” evaluation methods may not be related to the patients' opinion about outcomes.

We conclude that patients having surgery for ulnar nerve compression at the elbow, at a tertiary referral hospital, show a great variation in symptoms and surgical outcomes as well as extensive comorbidity, such as other nerve compression lesions as well as neck and shoulder pain. We suggest that complex cases should continue to be referred to the department of hand surgery, not only for surgery but also for the sometimes demanding postoperative care of the patient. Simple decompression seems to be a reliable first choice for surgery because of the relatively low level of complications and acceptable surgical outcome. Ulnar nerve transposition can be used in selected cases or when simple decompression fails to reduce the patient's symptoms. Low age seems to prompt the need for revision surgery with transposition, while higher age and female gender, respectively, are associated with greater dissatisfaction and poorer outcomes measured by DASH. All patients should be strongly recommended to stop smoking considering the remarkably increased risk of complications among smokers.

## Figures and Tables

**Table 1:** Reported pre- and postoperative symptoms in cases with surgery for ulnar nerve compression.

		Simple decompression			Primary anterior subcutaneous transposition			Secondary anterior subcutaneous transposition			*p* value
		Mean (SD)	Percentage	Count (*n*)	Mean (SD)	Percentage	Count (*n*)	Mean (SD)	Percentage	Count (*n*)	
VAS-estimated pain at rest preoperatively		5.2		56	5.5		29	5.4			0.84
		(2.8)			(3.1)			(2.3)			

VAS-estimated pain during activity preoperatively		5.8		56	7.4		29	6.8		14	**0.03**
		(2.8)			(2.4)			(2.7)			

McGowan grade preopereatively	Grade 1		13	8		10	3		7	1	0.32
	Grade 2		44	28		13	4		21	3	
	Grade 3		43	27		77	24		71	10	

Occurrence of paraesthesia preoperatively	Never		0	0		13	4		7	1	0.93
	Sometimes		10	6		6	2		7	1	
	Often		45	28		28	9		43	6	
	Always		45	28		53	17		43	6	

Experience of reduction in sensation preoperatively	Never		15	9		9	3		0	0	0.75
	Sometimes		16	10		28	9		43	6	
	Often		37	23		16	5		29	4	
	Always		32	20		47	15		29	4	

Occurrence of claw hand deformity preoperatively	Yes		71	18		47	15		36	5	0.23
	No		29	44		53	17		64	9	

Experience of reduction in grip strength preoperatively	Not at all		12	7		10	3		14	2	**0.004**
	Mild		23	14		7	2		7	1	
	Moderate		43	26		23	7		14	2	
	Pronounced		23	14		60	18		64	9	

Experience of reduction in ability to abduct or adduct fingers preoperatively	Not at all		47	28		27	8		43	6	0.09
	Mild		25	15		20	6		21	3	
	Moderate		12	7		23	7		14	2	
	Pronounced		17	10		30	9		21	3	

Difficulty sleeping because of arm/hand preoperatively	Never		15	9		16	5		7	1	0.58
	Sometimes		19	12		9	3		36	5	
	Often		44	27		44	14		36	5	
	Always		23	14		31	10		21	3	

VAS-estimated pain at rest postoperatively		2.8		56	2.8		29	3.1		14	0.12
		(2.6)			(2.5)			(2.1)			

VAS-estimated pain during activity postoperatively		3.7		55	4.5		29	4.6		14	0.33
		(2.7)			(3.0)			(2.6)			

Occurrence of paraesthesia postoperatively	Never		4	4		16	5		7	1	0.99
	Sometimes		45	27		38	12		43	6	
	Often		27	16		16	5		36	5	
	Always		22	13		31	10		14	2	

Experience of reduction in sensation postoperatively	Never		31	19		31	10		14	2	0.75
	Sometimes		31	19		28	9		36	5	
	Often		15	9		16	5		36	5	
	Always		23	14		25	8		14	2	

Experience of reduction in grip strength postoperatively	Not at all		26	16		13	4		7	1	**0.048**
	Mild		27	17		26	8		21	3	
	Moderate		36	22		36	11		43	6	
	Pronounced		11	7		26	8		29	4	

Experience of reduction in ability to abduct or adduct fingers postoperatively	Not at all		58	35		45	14		50	7	0.28
	Mild		18	11		19	6		29	4	
	Moderate		18	11		13	4		7	1	
	Pronounced		5	3		23	7		14	2	

Difficulty sleeping because of arm/hand postoperatively	Never		31	19		28	9		15	2	0.60
	Sometimes		25	28		50	16		54	7	
	Often		7	8		13	4		23	3	
	Always		5	6		9	3		8	1	

The Kruskal–Wallis test was used for all the variables, except for VAS- (visual analogue scale-) estimated pain at rest and during activity and preoperative McGowan grade, where one-way ANOVA and chi-square statistics were applied, respectively. Statistically significant differences are marked in bold. Values are mean (SD), percentage, and count *n*.

**Table 2:** Association between McGowan grade and postoperative patient-rated satisfaction.

	Dichotomised patient-rated satisfaction	
	Pleased, *n* (%)	Displeased, *n* (%)
McGowan grade 1	7/12 (58)	5/12 (42)
McGowan grade 2	20/35 (57)	15/35 (43)
McGowan grade 3	27/55 (49)	28/55 (51)

No association was found using chi-square statistics (*p*=0.62).

**Table 3:** Background data for 202 cases with surgery for ulnar nerve compression at the elbow.

		Simple decompression			Primary anterior subcutaneous transposition			Secondary anterior subcutaneous transposition			*p* value
		Mean (SD)	Percentage	Count (*n*)	Mean (SD)	Percentage	Count (*n*)	Mean (SD)	Percentage	Count (*n*)	
Age at the time of the first surgery		51 (13)			48 (14)			44 (14)			**0.01**
Gender	Female		59	67		64	36		72	23	0.38
Smoking	Smoker		29	33		34	19		34	11	0.76
Neck problems	Yes		26	30		27	15		16	5	0.43
Shoulder problems	Yes		12	14		21	12		22	7	0.21
Other neuropathy in the same arm	Yes		56	64		55	31		63	20	0.78
Other neuropathy in the opposite arm	Yes		38	43		54	30		53	17	0.08
Diabetes	Yes		12	13		13	7		20	6	0.49
Electrophysiology for ulnar nerve affection at the elbow level	Yes		61	69		45	25		56	18	0.09^a^
	No		28	32		45	25		31	10	
	Not performed		10	11		11	6		13	4	
MRI for signs of nerve root affection	Yes		15	17		14	8		12	4	0.46^a^
	No		26	30		43	24		41	13	
	Not performed		59	67		43	24		47	15	

Differences were evaluated with chi-square statistics, except for “age at the time of the first surgery,” where one-way ANOVA was used. Statistically significant differences are marked in bold. Values are mean (SD), percentage, and count (*n*). MRI: magnetic resonance imaging. ^a^*p* values for electrophysiology and MRI are based on the examinations evaluating affection of the ulnar nerve and spinal nerve root(s), respectively.

**Table 4:** Occurrence of complications in 202 cases having surgery for ulnar nerve compression at the elbow.

	All patients, *n* (%)	Simple decompression, *n* (%)	Primary anterior subcutaneous transposition, *n* (%)	Secondary anterior subcutaneous transposition, *n* (%)
Total	205^a^			
None	164/202 (81)	102/114 (89)	38/56 (68)	23/32 (72)
Emergent neurogenic pain after surgery	16/202 (8)	2/114 (2)	10/56 (18)	4/32 (13)
Affection of nerve function^b^	15/202 (7)	7/114 (6)	7/56 (13)	1/32 (3)
Postoperative infection	6/202 (3)	3/114 (3)	2/56 (4)	1/32 (3)
CRPS	4/202 (2)	1/114 (1)	0/56 (0)	3/32 (9)

^a^Three of the patients had two different types of complications making the total number 205. ^b^Affection or loss of nerve function, including hypoesthesia, around the area of surgery. CRPS: complex regional pain syndrome.

## Data Availability

The data used to support the findings of this study are available from the corresponding author upon request.

## Supplementary Materials

Supplementary Materials Appendix 1: a specially designed questionnaire sent out by mail to patients having surgery for ulnar nerve compression.
