# Supplementary material for: Patient Characteristics in Ulnar Nerve Compression at the Elbow at a Tertiary Referral Hospital and Predictive Factors for Outcomes of Simple Decompression versus Subcutaneous Transposition of the Ulnar Nerve
Source: Biomed Res Int. 2019 Dec 19;2019:5302462. doi: 10.1155/2019/5302462 (PMC6948284; doi:10.1155/2019/5302462)
Supplement: Supplementary Materials — Appendix 1: a specially designed questionnaire sent out by mail to patients having surgery for ulnar nerve compression. [file 5302462.f1.pdf]

Code number:

Questionnaire for evaluation of results after surgery due to ulnar nerve compression

Circle the option which fits you the best!

1. Can you perform your professional work after the surgery concerning the hand/arm?

   Yes *Partially* *No*

2. Do you have to refrain from any activity after the operation due to pain/ache/weakness in the operated area?

   Yes *Partially* *No (not at all)*

   Comments (e.g. if something has become more difficult for you to do?) …………..

   ……………………………………………………………………………………..

3. Are you ***right-handed*** or ***left-handed***?

   Right *Left*

4. How do you think the arm/hand works **today** compared to before the surgery?

   Completely fine *Better* *No difference* *Worse*

5. Were you troubled with numbness/tingling in the fingers/hand **before** surgery?

   Always *Often* *Sometimes* *Never*

   If your answer is always, often or sometimes, which fingers, or parts of the hand were affected?

   ……………………………………………………………………………………

6. Are you experiencing numbness/tingling in the fingers/hand **after** the surgery?

   Always *Often* *Sometimes* *Never*

   If your answer is always, often or sometimes, which fingers, or parts of the hand are affected?
   ……………………………………………………………………………………….

7. Did you experience any reduction in sensation in the fingers/hand **before** the surgery?

   Always *Often* *Sometimes* *Never*

   If your answer is always, often or sometimes, which fingers, or parts of the hand were affected?
   ………………………………………………………………………………………

8. Do you experience any reduction in sensation in the fingers/hand **after** the surgery?

   Always *Often* *Sometimes* *Never*

   If your answer is always, often or sometimes, which fingers, or parts of the hand are affected?

   ……………………………………………………………………………………….

9. Did you experience any reduction in grip strength of the hand **before** surgery?

   Not at all *Mild* *Moderate* *Pronounced*

10. Do you experience any reduction in grip strength of the hand **today**?

    Not at all *Mild* *Moderate* *Pronounced*

11. Did you experience any reduction in the ability to abduct or adduct the fingers **before** surgery?

    Not at all *Mild* *Moderate* *Pronounced*

12. Do you experience any reduction in the ability to abduct or adduct the fingers **today**?

Not at all *Mild* *Moderate* *Pronounced*

13. Were you troubled with "claw hand deformity" of the ring finger and little finger **before** surgery? (see picture)

Yes *No*

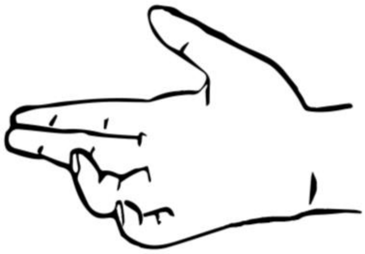

14. Are you troubled with "claw hand deformity" of the ring finger and little finger **today**? (see picture)

Yes *No*

15. Did you smoke at the time of the surgery?

Yes *No*

16. Did you have any trouble sleeping because of the arm/hand **before** the surgery?

Always *Often* *Sometimes* *Never*

17. Do you have any trouble sleeping **today** because of the arm/hand?

Always *Often* *Sometimes* *Never*

18. Do you have any other disease/injury in another nerve or in the arm, and if so, which one/ones?

……………………………………………………………………………………….

19. Do you have any other disease/diagnosis, and if so, which one/ones?

……………………………………………………………………………………...

20. Are you taking any drugs regularly, and if so, which one/ones?

……………………………………………………………………………………...

21. For how long did you have symptoms before the surgery? …………………………..

22. Are you pleased with the results of the surgery?

Very pleased *Generally pleased* *A bit displeased* *Very displeased*

Comment (e.g. if you want to express how pleased or displeased you are) ………….

……………………………………………………………………………………...

23. Based on what you know today about the surgery and the period afterwards, would go through the same procedure again?

Yes *Not sure* *No*

Comment:…………………………………………………………………………

…………………………………………………………………………………..…

Self-reported pain (VAS)

1. Can you estimate your pain by making a mark on the line below?

   a) At rest **before** the surgery?

   No pain I--I Worst possible pain

   b) During activity **before** the surgery?

   No pain I--I Worst possible pain

   c) At rest **after** the surgery?

   No pain I--I Worst possible pain

   d) During activity **after** the surgery?

   No pain I--I Worst possible pain
